# Prebiotic Potential of Dietary Beans and Pulses and Their Resistant Starch for Aging-Associated Gut and Metabolic Health

**DOI:** 10.3390/nu14091726

**Published:** 2022-04-21

**Authors:** Saurabh Kadyan, Aditya Sharma, Bahram H. Arjmandi, Prashant Singh, Ravinder Nagpal

**Affiliations:** Department of Nutrition and Integrative Physiology, College of Health and Human Sciences, Florida State University, Tallahassee, FL 32306, USA; sk21bq@my.fsu.edu (S.K.); al21bl@my.fsu.edu (A.S.); barjmandi@fsu.edu (B.H.A.); psingh2@fsu.edu (P.S.)

**Keywords:** aging, beans, fiber, gut health, lentils, microbiota, microbiome, prebiotic, pulses, resistant starch

## Abstract

Dietary pulses, including dry beans, lentils, chickpeas, and dry peas, have the highest proportion of fiber among different legume cultivars and are inexpensive, easily accessible, and have a long shelf-life. The inclusion of pulses in regular dietary patterns is an easy and effective solution for achieving recommended fiber intake and maintaining a healthier gut and overall health. Dietary pulses-derived resistant starch (RS) is a relatively less explored prebiotic ingredient. Several in vitro and preclinical studies have elucidated the crucial role of RS in fostering and shaping the gut microbiota composition towards homeostasis thereby improving host metabolic health. However, in humans and aged animal models, the effect of only the cereals and tubers derived RS has been studied. In this context, this review collates literature pertaining to the beneficial effects of dietary pulses and their RS on gut microbiome-metabolome signatures in preclinical and clinical studies while contemplating their potential and prospects for better aging-associated gut health. In a nutshell, the incorporation of dietary pulses and their RS in diet fosters the growth of beneficial gut bacteria and significantly enhances the production of short-chain fatty acids in the colon.

## 1. Introduction

Pulses are valuable dry grains from leguminous crops. Domesticated around 10,000 years ago, pulses have been consumed as a key staple food crop, especially in developing nations, thus providing a primary means of protein and energy [1]. However, the past century has witnessed a change in the eating habits of the population, especially the decline of the pulse consumption in the daily diet and a surge in the chronic disease rates [2]. Based on a posteriori and a priori dietary patterns, consumption of whole grains and legumes/pulses are linked with longevity and better cardiovascular, metabolic, and cognitive health [3]. On the contrary, diets rich in refined grains, red meat, and sugar have been associated with an increased risk of mortality and adverse cardiometabolic outcomes [3].

Although there are numerous pulse varieties available worldwide, Food and Agriculture Organization (FAO) has listed 11 main types, namely beans, broad beans, Bambara beans, chickpeas, lentils, cowpeas, peas, pigeon peas, vetches, lupins, and other “minor” pulses [4]. Among them, lentils (*Lens culinaris* L.), beans (*Paseolus vulgaris* L.), chickpeas (*Cicer arietinum* L.), and peas (*Pisum sativum* L.) are the most frequently consumed pulses worldwide [5]. Pulses possess superior nutritional properties and harbor various bioactive compounds, viz., fermentable fibers, bioactive peptides, and phytochemicals [6]. The high nutritional value of pulses is attributed to their high-quality protein and soluble and insoluble dietary fiber [7]. The daily intake of dietary fiber at a level of 14 g/1000 kcal or above has been proposed to confer health benefits in human cohorts [8]. Still, a developed nation like the United States is far from achieving this level, and the magnitude of the gap is nearly 50–70% shortfall [9]. To address this shortfall, supplementation of diets with pulses could be one promising strategy as the total fiber content in pulses can range up to 30 g/100 g dry weight (peas: 14–26 g; lentils: 18–20 g; chickpeas: 18–22 g; beans: 23–32 g), with insoluble fiber being the major sub-component (peas: 10–15 g; lentils: 11–17 g; chick-peas: 10–18 g; beans: 20–28 g) [4].

Starch is the major carbohydrate in pulses accounting for nearly 50% portion of carbohydrates [10]. Certain starches present in the raw and/or cooked pulses exist in the form of dietary fiber instead of available carbohydrates. This is due to the partial or complete modification in the starch structure during heat processing of foods leading to the formation of resistant starch (RS). RS remains un-digested in the upper-gastrointestinal tract and reaches the large intestine, where it is metabolized by intestinal microbes into a wide range of metabolites, which helps in the maintenance of optimal human health [11]. Past studies have also proven the prebiotic potential of RS in improving the post-prandial glycemic and insulinemic responses, increasing satiety, reducing cholesterol and stored fat, and promoting weight loss, making it an apt ingredient, especially for the management of gut-associated metabolic disorders [12,13,14,15]. Hitherto, studies assessing the human health benefits of RS were confined to RS derived from cereals and tubers, with little to no focus given on RS derived from pulses. Recently, efforts were made in our lab to isolate and purify starches from 18 pulses which were evaluated for their functional properties in order to promote their use as superior food ingredients in industry [16]. Owing to the superior sensory property of selected pulse RS compared to traditional fibers like whole cereals, fruit fibers, etc., the supplementation of this functional ingredient in diet could act as a beneficial nutritional intervention for the control of metabolic diseases [17].

Nowadays, it has been widely popularized that the human gastrointestinal (GI) tract is a frontline mediator system wherein the intestinal bacteria aid in the digestion of dietary constituents of consumed foods and synthesizes low molecular weight bioactive molecules, which ultimately exerts a crucial role on human health and well-being [18]. The human gastrointestinal tract contains nearly 10^14^ microorganisms belonging to over 1000 species and has a bacterial genomic content of approximately 100 times over compared to the human genome [19]. About 95% of the total microbes present in the human body are colonized in the GI tract. The GI tract is the home of bacteria, eukaryotes, and archaea and is collectively known as gut microbiota [20]. Several factors such as the morphology of the gut, nutrient availability, pH, and presence or absence of oxygen are responsible for the variation in gut microbiota composition and growth of certain microbial taxa specific to different regions of the gut. The most common gut bacteria are associated with the four major phyla, with the most abundant being Firmicutes (65%), followed by Bacteroidetes (25%), Proteobacteria (8%), and Actinobacteria (5%). Moving down the taxonomic hierarchy, the GI tract harbors three main groups of extremophile anaerobes *Clostridium coccoides* group (or *Clostridium cluster XIVa*), *Clostridium leptum* group (or *Clostridium cluster IV*), and *Bacteroides* [21]. The gut microbes, together with their metabolites produced as a result of the degradation of different substrates, provide a range of immune, metabolic and neurobehavioral functions to host health. 

Gut microbiota is dynamic in nature and changes continuously during the lifespan of an individual [22]. During the aging process of an individual, dynamic changes occur in behavioral, environmental, biological, and social processes. Genomic instability, epigenetic alterations, and telomere attrition are primary indicators of aging, resulting in cellular senescence, problems in nutrient sensing, and mitochondrial-related dysfunctions, which further negatively impact intercellular communication and exhaustion of stem cells [23]. Thus, the aging-associated decline in the cellular functions and immune system responses leads to chronic low-grade inflammation and increased gut permeability, thereby marking the onset of various gastrointestinal disorders, cardiometabolic disease, muscle frailty, cognitive decline, and gut dysbiosis [24]. Aging-associated problems are further aggravated by the ill effects of western diets rich in fat and sugars, which may increase the propensity towards gut dysbiosis [25]. The maintenance of a healthy and diverse gut microbiota that coevolves with our lifespan is a principal factor in the amelioration of various age-related diseases. Earlier studies by our group indicated that the severity of gut dysbiosis is higher in older cohorts than the young ones [26]. 

Over 50% of ‘baby boomers’ are considered at nutritional risk, and this statistic could grow to over 30% by 2050, indicating the economic burden on the American health care system while underscoring the significance of nutrient-dense, health-promoting food sources as a preventive strategy. To this end, pulses could be a ‘perfect’ food choice for older adults as they have a higher fiber and protein contents, and low glycemic index (and low saturated fat) and are easy to buy, prepare, and consume, thereby offering an inexpensive way to specifically promote gut health and overall health of all age groups including older subjects [27]. Gut health refers to a symbiotic relationship of the host immune system with a balanced gut microbiota to preserve the integrity of functional intact mucosal epithelial barrier and to reduce adverse inflammatory responses [28]. The disturbance in this relationship due to gut microbiota dysbiosis leads to the advancement of various chronic gut-related diseases like obesity, inflammatory bowel disease (IBD), colorectal cancer, and diabetes [29]. Gut dysbiosis is mostly characterized by reduced diversity (species richness) of commensal and beneficial gut microbes with concomitant overgrowth and/or proliferation of indigenous pathobionts or opportunistic pathogenic microbes, thereby triggering immune dysregulation and a state of a low-grade pro-inflammatory reaction in the gut [30]. This perturbed (dysbiotic) balance in the bidirectional cross-talk between gut microbiota and epithelial immune system further aggravates intestinal epithelial (altered gut barrier function; ‘leaky gut’), immunological (chronic hyper-inflammation of intestinal mucosa) and neurological (gut–microbiota–brain axis) dysfunctions leading to the development of various gut-related and systemic diseased states [31]. In these contexts, this review aims to collate information on understanding the influence of dietary pulses and their RS consumption on the shifts in the gut microbiome and metabolome profile in different cohorts and their associated health outcomes. In addition, special focus is given to the existing literature examining the impact of RS on aging-associated gut and metabolic health.

## 2. Resistant Starch and Human Health

Starch is a dietary carbohydrate that is commonly found in everyday food. It is the second most abundant chemical compound in the plants after cellulose. Chemically, starch is composed of two monosaccharide molecules that are amylose (linear chain) and amylopectin (branched chain). These molecules are linked together with alpha 1-4 and/or alpha 1-6 glycosidic bonds. Based on physical and physiological properties, starch can be classified into three categories, namely rapidly digestible starch, slowly digestible starch, and resistant starch (RS) [32]. Englyst and coworkers (1982), in an in vitro study, found that some portion of the starch remained undigested even after enzymatic treatment. Further studies confirmed that these starches were undigested by the amylases in the small intestine and enter the colon, where it is utilized by gut microbial communities. They named this starch fragment “resistant starch” [33]. The digestibility of the starch in the small intestine is primarily affected by the structure of the starch molecule and the ratio of amylose to amylopectin. Chemically, RS has a relatively low molecular weight (12 KDa) and has a linear structure made up of α-1,4-D-glucan moieties obtained from the retrograded amylose fraction [17].

Resistant starch is further subdivided into five types depending upon its structural features. RS type 1 (RS1) is physically inaccessible starch and has the most complex structures as it is frequently found entrapped within protein matrix or non-starch components of the plant cell wall (e.g., whole grains or pulses) [11]. Compared to RS1, the cellular structure is absent in RS type 2 (RS2). The RS type 2 possesses native, uncooked, and semi-crystalline starch granules having a B- or C-type polymorph (e.g., high-amylose starch, raw potato starch) [11]. The RS type 3 (RS3) is obtained by retrogradation process upon cooking and cooling of starch-containing foods. Its resistance to digestion could be due to lower activity of pancreatic α-amylases toward starch double helices as against fully gelatinized starch molecules (e.g., retrograded high amylose maize starch) [34]. The RS type 4 (RS4) is the starch-modified through chemical processes such as esterification, crosslinking, hydroxypropylation, acetylation, and phosphorylation [35]. The functional groups block the site of action of starch digestive enzymes, which confers resistance of RS4 to digestion. The RS type 5 (RS5) is defined as the starch obtained by complex formation between high amylose starch with the lipids, which further increases the enzyme resistance of high amylose by preventing granule swelling during cooking [17]. 

**Figure 1:**
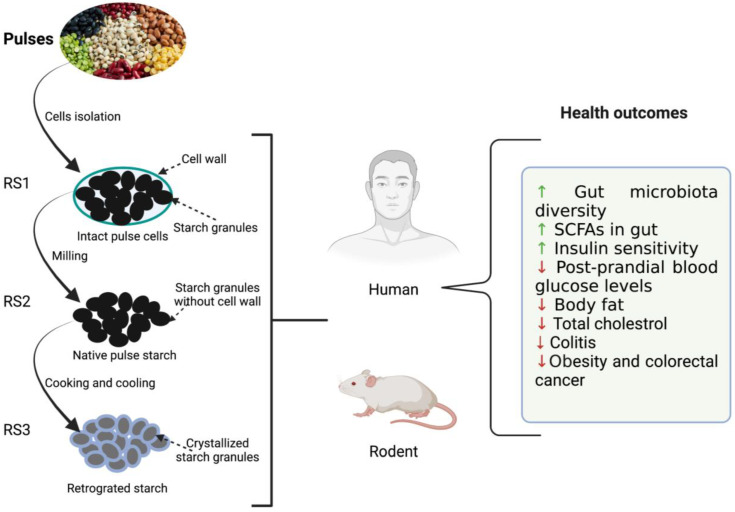
Illustration depicting the effects of resistant starches (RS) derived from dietary beans and pulses on host (rodent and human) health. ↑: increased; ↓: decreased.

Resistant starch possesses many desirable functional and health-promoting properties [32]. An overview of the effect of resistant starch derived from the pulses on the health outcome of humans and rodents is summarized in Figure 1. RS fermentation in the lower GI tract produces different starch oligomers and SCFAs. SCFAs are actively involved in reducing the risk of diabetes, cancer, obesity, and other cardiovascular diseases [8,19,25]. Among them, acetate, propionate, and butyrate have been extensively studied for their health benefits. Acetate is the major SCFA that is produced to the tune of 65% in the colon resulting in significant drops in pH. Thus, it helps in the inhibition of various pathogenic microorganisms and indirectly aids in the absorption of minerals such as calcium, iron, and sodium Butyrate, on the other hand, provides energy to colonocytes, possesses anti-inflammatory properties, protects against colon cancer, and plays a key role in gut homeostasis as well as maintaining the integrity of epithelium [36]. Butyrate is also responsible for lower levels of glycolysis and glycogenolysis (Ashwar et al., 2017). Propionate is another important metabolite that is partially absorbed via portal veins and reaches the liver. It is then metabolized as a glucogenic substrate resulting in inhibition of pathways leading to reduced 3-hydroxy-3-methylglutaryl co-enzyme A (HMG-CoA) activity and suppression of acetyl-CoA reductase, thereby imparting reduction in blood plasma cholesterol levels [37]. The serum cholesterol-lowering effect of RS was demonstrated in rats when they were fed a cholesterol-free diet [38]. 

## 3. Utilization of Resistant Starches by Gut Microbiota

A diverse community of microbes present in the gut degrades the dietary fiber, including resistant starch in the colon. The human genome encodes only 17 enzymes to metabolize food glycans viz., starch, sucrose, and lactose [39], while a wide range of fibers can be utilized by gut microbial enzymes consisting of 130 glycoside hydrolase (GH), 22 polysaccharides lyase [7] and 16 carbohydrate esterase (CE) [40]. Dobranowski and Stintzi [41] discussed the RS degradation by gut microbes and divided the degraders into three main categories, namely primary degraders, secondary degraders, and cross feeders. RS utilization by primary degraders is influenced by the starch granules when they are grown in monoculture. Primary degraders penetrate the intact granule structure by initiating their catalytic action on the outer granule surface. As a result, there is liberation of oligosaccharides along with some metabolites, such as acetate and lactate [42]. The best primary degraders are found to be *Bifidobacterium adolescentis* and *Ruminococcus bromii*. These species unmask the resistant starch with their complex enzymatic action. *Ruminococcus bromii* is an important part of the gut community, present to the tune of 3% of gut microbiota [43]. Five strains of *R. bromii* contain 17 GH-13 amylases, which catalyzes the hydrolysis of alpha 1,4 and alpha 1,6 glycosidic linkage [44]. It forms several by-products while degrading starch, namely glucose, maltose, and oligosaccharides, and also liberates ethanol, propanol, acetate, and formate [45]. Recently, a new *Ruminococcus* species FMB-CY1 was identified, having a close resemblance to *Ruminococcus bromii* [46]. This species is able to degrade commercial resistant starch of types 2, 3, and 4 into simple carbohydrates (glucose and maltose). Jung et al. [47] demonstrated utilization of RS by 2 out of 15 strains of *Bifidobacteria adolescentis*. After fermenting RS, *B. adolescentis* liberates acetate, lactate, and formate and is able to utilize more starch by-products when compared with *R. bromii* (Belenguer et al., 2006). 

Secondary degraders can degrade regular starch due to the presence of amylases but poorly utilize resistant starch, or in some cases, they are unable to degrade RS. They can grow on RS in monoculture similar to primary degraders. However, they readily utilize starch by-products (oligosaccharides) that are generated by other degraders. For efficient working, they require primary degraders to act on the smooth surface of the resistant starch granule. The eroded surface is suitable for their attachment. Typically, secondary degraders align themselves near to the primary degraders and utilize their excess by-products (Dobranowski and Stintzi, 2021). Secondary degraders consist of *Eubacterium rectale*, *Roseburia*, *Butyrivibrio*, *Bacteroides thetaiotaomicron*, and *Bifidobacteria. E. rectale* is a key member of the bacterial community (i.e., *Clostridium XIVa*), which generates butyrate and helps in maintaining homeostasis of the gut. The amylopectin hydrolysis capacity of this bacteria is twice compared to amylose (Lopetuso et al., 2013). Butyrogenic species such as *Roseburia faecis* utilize amylopectin more readily but amylose poorly [48]. *Bacteroides thetaiotaomicron* is capable of degrading different types of glycans (at least 32) effectively [49]. Further, it is also associated with the production of acetate, lactate, and propionate; however unable to synthesize butyrate [50]. Several species of *Bifidobacteria*, e.g., *B. infantis*, *B. longum*, *B. bifidum*, and *B. breve*, could act as secondary degraders. In addition, improved growth of *B. cuniculi* and *B. magnum* on starch have been found when they are co-cultured [51]. 

Cross feeders cannot directly metabolize starch and are unable to grow in monoculture. These microbes play an important role in the conversion of upstream by-products and metabolites [41]. Starch by-products generated by primary degraders such as lactate, formate, and succinate are utilized by cross feeders. They help in maintaining overall fermentation and desired equilibrium among gut microbes. The entire ecosystem is supported by the produced metabolites and sequential cross-feeding mechanisms, which are mostly acidic in nature [42]. For example, *R. hominis* cannot utilize starch, but when co-cultured with *B. adolescentis*, they grow well on by-products (lactate and acetate), which are generated by *B. adolescentis*. In addition, *R. hominis* only utilize malto-oligosaccharide and cannot degrade amylose and amylopectin [48]. 

Further, the structural difference in the RS can affect the metabolizing capacity of gut bacteria. It has also been observed that the growth of some gut bacteria is differentially upregulated based on RS types. In a human study, at the phyla level, RS4 consumption increased the abundance of *Bacteroidetes* and *Actinobacteria*, while decreasing the prevalence of Firmicutes as compared to RS2 [52]. At the species level, the abundance of *Bifidobacterium adolescentis* and *Parabacteroides distasonis* was promoted after RS4 consumption, while RS2 promoted the abundance of *Eubacterium rectale* and *Ruminococcus bromii* [52]. In a pig study fed on RS3, the relative abundance of *Faecalibacterium prausnitzii* was increased while the number of *Escherichia coli* and *Pseudomonas* spp. decreased [53]. Furthermore, RS3 having a B-type crystalline structure which favors the growth of *Bifidobacterium* spp., whereas RS3 with A-type polymorphic form enriched the prevalence of *Atopobium* spp. [54]. Hence, there is a need for detailed structural characterization of raw RS present in different legume cultivars, as well as conformational changes in RS induced by different processing treatments in order to precisely target the gut microbiota modulation.

## 4. Prebiotic Characteristics of Dietary Resistant Starches

Prebiotics are non-viable food ingredient which are selectively metabolized by beneficial gastrointestinal microbiota thereby inducing specific changes in the microbiota composition and/or activity, thus conferring benefit(s) upon host health [55]. Dietary carbohydrates have become a potential prebiotic candidate. Dietary carbohydrates such as resistant starch, hemicellulose, sugar alcohols including maltitol, lactitol, and sorbitol, soybean oligosaccharides, lactosucrose, gluco-oligosaccharides, isomalto-oligosaccharides, gentio-oligosaccharides, xylooligosaccharides, polydextrose, lactose, b-glucans, resistant dextrins, oligosaccharides from melibiose, oat bran, N-acetylchitooligosaccharides, and mannan-oligosaccharides have been studied in the past for their prebiotics benefits [55,56]. To be considered as a prebiotic, resistant starch must fulfill the following criteria: resistance to upper GI digestive enzymes and gastric acidity; fermentation by gut microbiota; and foster the growth of specific health-promoting bacteria [55]. These three criteria are used further to understand the potential resistant starch as prebiotics. 

(i) Resistance to digestive enzymes and gastric acidity: Due to complex physico-chemical properties and structural characteristics of resistant starch, a specific value of resistance cannot be developed. Naturally occurring resistant starches, i.e., RS2 and RS3, are inaccessible to digestive enzymes present in the gut mainly due to their structure. Starch modification (RS3 and RS4) affects the resistance against gastric acidity. Resistance of the starch also depends on the amylose to amylopectin ratio. These molecules are arranged in semi-crystalline form and provide integrity and stability to the starch granules. Li et al. [57] reported that the digestibility of starch by enzymes decreases as the amylose to amylopectin ratio increases. In addition, the starch digestibility can be negatively influenced by lipid content because their interaction results in lipid amylose complex (RS5), which prevents starch swelling [58]. 

(ii) Fermentable by the gut microbiota: Starch, after resisting the harsh condition of the digestive tract, finally enters the colon part of the human body, where it is utilized by a wide array of gut microbes. Bacteria responsible for starch fermentation can be characterized into two types, namely proteolytic and saccharolytic bacteria. Proteolytic bacteria act on the protein structures, and saccharolytic bacteria break down the carbohydrate molecules [56]. 

(iii) Foster the growth of health-promoting bacteria: Dietary fibers are fermented by a wide range of gut microbiota, such as Bifidobacteria, Clostridia, Bacteroides, and Lactobacilli. Complex microbiological techniques are used to quantify the increased abundance of gut microbes. Selective quantification of target bacteria using molecular techniques (e.g., real-time PCR) and assessment of change in entire gut bacterial composition relative to baseline via metagenomics approach are considered reliable tools for estimating the effects of RS treatment. Further, measuring the increased production of organic acids and gas can be indirectly associated with the significant growth of the bacteria community in the gut [56].

## 5. Benefits of Dietary Beans and Pulses on Gut Health

The recent advances linking the role of dietary fibers in ameliorating different disease states have led to increased interest in pulse-based foods. Various types of fibers present in pulses include long-chain soluble and insoluble polysaccharides, resistant starch, and galactooligosaccharides. In addition, these components can act as prebiotic precursors, which are digested by beneficial microorganisms in the gut. The consumption of pulses in the diet has been linked to the reduction in serum cholesterol, increased satiety, and low post-prandial blood glucose levels, thus mitigating the risk of different metabolic diseases like cardiovascular diseases, obesity, diabetes, etc. [59,60]. In fact, several meta-analyses concluded that daily pulse intake of approximately 2/3 cups could significantly lower total and LDL cholesterol [61]. The low glycemic response of pulse is associated to the physical barrier between the starch and digestive enzymes by the intact cell wall of whole pulses after cooking. Furthermore, pulse consumption is closely associated with reducing blood pressure and providing protection against reactive oxygen species due to the presence of high levels of polyphenols [62].

In the last few years, more research has been directed towards pulses which could be a sustainable source of plant protein compared to animal protein to feed the growing population and to simultaneously address the food insecurity problems [4]. Additionally, whole pulses being rich in plant-based protein and dietary fibers underpins the hypothesis of their positive effects on the gut microbiota. Table 1 summarizes the influence of consumption of pulses in various forms—cooked, flour, meals, or supplemented in the diet, on the gut microbiota changes in rodents and humans. A study on pulse flour exhibited improved growth of genera *Bifidobacterium*, *Faecalibacterium*, *Clostridium*, *Eubacterium*, and *Roseburia* along with enhanced butyrate and acetate production [63]. Several studies have reported that the incorporation of pulses in the diet increases the abundance of *Prevotella*, *Dorea*, and *Ruminococcus flavefaciens*, and decreased abundance of *Ruminococcus gnavus* in mice models [18,29,64,65,66]. *Prevotella* is a genus possessing a large spectrum of glycoside hydrolases and is known for its ability to produce SCFAs following the carbohydrates fermentation [29]. The species *Ruminococcus flavefaciens* had been found to decrease in overweight (BMI: 25.0–29.9) and obese (BMI: >30.0) subjects [67]. The abundance of *Ruminococcus gnavus*, a mucolytic species, has been linked to an increase in gut-barrier pathologies in subjects with obesity and inflammatory bowel disease [65]. Another positive effect of pulse intake is the increased prevalence of *Akkermansia muciniphila* in the gut, which is often categorized as next-generation probiotics [8,68]. Interestingly, this bacterium is also mucolytic but has an inverse correlation with *R. gnavus* [69]. Majority of the studies reported herein demonstrated a decrease in the ratio of Firmicutes to Bacteroidetes. This reduction in the ratio of two major phyla has been associated with the amelioration of obesity, possibly due to altered energy extraction from carbohydrates metabolism in the colon [70]. Among the Bacteroidales, the members representative of the pulse-based diets includes *Muribaculaceae* (*S24-7*), *Rikenellaceae* and *B. acidifaciens* [18]. Lentil consumption was found to be associated with increased prevalence of *Roseburia* in mouse feces [64]. *Roseburia* is involved in butyrate production and has negative correlation with several diseases such as colitis and Crohn’s disease [71]. Although these studies revealed beneficial effects of pulses in positively modulating the gut microbiome, the impact on different gut genera is complex, which may be dependent upon many variables, such as pulse type, dose, age, status of cohorts, duration of the study and the sequencing methodology adopted. 

Common beans, chickpea, and lentils have been shown to exert positive effects in the modulation of the colonic microenvironment in animal models [18,29,64,66]. These include enhancement of (i) crypt mucus content and mucin mRNA expression; (ii) expression of epithelial tight junction proteins; (iii) crypt length, epithelial cell proliferation, and goblet cell number; (iv) SCFAs levels (acetate, propionate, and butyrate); (v) expression of G protein-coupled receptors in the intestine; (vi) reduced pro-inflammatory cytokines in the serum. Increased expression of G protein-coupled receptors in the colon is related to sensing high SCFA production by gut microbes which are implicated in adipose tissue metabolism and appetite regulation [81]. The benign role of whole pulse consumption in the modulation of human gut microbiota and metabolite profile have also been explored in the past by researchers using clinical trials [72,73,74,75,76]. Some of these include reduction in pathogenic and putrefactive gut bacteria species; increase in *Bacteroidetes* and *Faecalibacterium prausnitzii*; decreased total serum cholesterol, LDL- and HDL-cholesterol; boost in microbial richness, and significant change in metabolite profile (e.g., ophthalmate) in colorectal cancer survivors.

## 6. Prebiotic Potential of Pulses-Derived Resistant Starch for Gut Health

The concentration of SCFAs in the lower GI tract normally reduces from the proximal to the distal colon. The amount of SCFAs production is majorly dependent upon the amount of fiber reaching the distal colon. Therefore, one way of increasing the SCFAs in the distal gut is the selection of dietary fibers, which are minimally digested prior to reaching the distal colon. Increasing the consumption of resistant starches in the diet is a promising strategy to modulate gut health and benefit the host. 

Native RS is present in varying proportions in cereals, tubers, and legumes. In addition, the RS content can be altered using cooking and cooling operations. Interestingly, the comparison of RS content among cooked cereals, legumes, and tubers samples showed legumes with the highest RS content [82]. Brummer, Kaviani and Tosh [6] reported that cooked pulses have a relatively high proportion of resistant starch (3.75–4.66% of pulse dry weight basis) than many other cooked foods. Similarly, Garcia-Alonso et al. [83] reported a marginal increase in the RS content of chickpeas, lentils, and common beans upon boiling, cooling, and reheating. Retrogradation of the gelatinized starch post-cooking and cooling is usually associated with the increased content of resistant starch in the cooked pulses [84]. Still, the amount of RS in raw, baked, and boiled pulses differ significantly, and it is a function of its intrinsic factors (e.g., amylose to amylopectin ratio, crystallinity, granular structure) and external factors (e.g., processing methods employed, storage period and conditions [13]. In brown lentils (*Lens culinaris*, Medikus), RS content was further increased by the addition of lipids, resulting in the formation of amylose-lipid complexes (RS5 type) [85].

Fermentation of resistant starch by the intestinal microbes in the distal gut brings about changes in the gut microbiota and metabolic profile. Table 2 summarizes the studies conducted recently on the impact of pulse-derived starch on the gut microbiome and metabolome. Mostly, these recent studies have started exploring the effect of pulse-based RS on humans through in vitro fecal fermentation studies [19,86,87,88], and very few studies have focused on rodent models [37,70]. 

Although ample clinical studies have been conducted in relation to changes in gut microbial community structure post-consumption of RS from cereal or tuber sources (Table 3), so far, to the best of our knowledge, no such attention is given towards clinical trials on pulse-derived RS.

A recent study conducted by Xu, Ma, Li, Liu and Hu [37] exhibited an increased abundance of *Anaerotruncus* and *Bacteroides* in mice fed with autoclaved retrograded lentil starch (RS3 type) relative to control and high-fat diet groups. *Anaerotruncus* is associated with the production of butyrate and/or propionate, while *Bacteroides* contribute to increased propionate production via the succinite pathway [108]. They also facilitate ameliorating oxidative stress and inflammation [37]. Another study on rats fed with a high-fat diet revealed a reduction in the weight gain and decreased abundance of *C. leptum* (cluster IV), a group known to increase in obese individuals [70].

Zhou, Ma and Hu [87] reported altered differences in the SCFA profile, which was dominated by propionate instead of acetate post-in vitro fermentation of pullulanase-debranched and acid-hydrolyzed pea starches [87]. Such divergent results could be attributed to inherent structural differences of semi-crystalline RS3 formed after debranching and acid hydrolysis than the native starch. As a result of this, there has been an increase in the abundance of some taxa differing from starch-degrading taxa [87]. Furthermore, some in vitro studies on RS reported reduced a-diversity and species richness/diversity [86,87]. Poor tolerance of some species such as *Bacteroides fragilis* to pH drop after fermentation could be one reason for reduced diversity [109]. Another possible speculation is related to the increased abundance of bacteriophages owing to the high availability of SCFAs post-fermentation, which might lead to a reduction in gut species composition and richness [110]. *Blautia* and *Roseburia* genera were found to increase post-fermentation of human fecal samples with RS derived from pinto beans and peas [19,87,88]. *Blautia* and *Roseburia* are members of the *Lachnospiracea* family associated with high butyrate production.

The production of SCFAs from RS fermentation is largely influenced by host health and diet, colonic environment, microbiota, and fiber’s structural characteristics [111]. Recently, a clinical study demonstrated that the discrete structure and structural features of RS play a crucial role in determining the shift towards either propionate or butyrate production during fermentation [105]. A study investigated the role of the intact and damaged structure of pinto bean cells on SCFA production during fecal fermentation [19]. The amount of SCFAs increased significantly after the enzymatic treatment of beans as compared to intact beans. Acetate and propionate production via the fermentation of various dietary fibers and RS is caused by bacteria belonging to Gram-negative Bacteroidetes phylum, while butyrate production is associated with bacteria associated with Firmicutes. *Bacteroides* occupy a large portion of Bacteroidetes, which have the inherent ability to ferment complex carbohydrates such as polysaccharides or RS. 

## 7. Resistant Starch in Context to Aging-Associated Health and Disease

Senescence is an inevitable and irreversible growth process dictated by the cascade of complex natural phenomena. Advances in research point towards a close connection between the ecology of intestinal flora and aging, and the intestinal ecological disorders could cause accelerated aging and shortening of lifespan [25]. Abnormal perturbations in the gut microbiome due to aging-related inadequate nutrition, illnesses, and medications lead to a state of ‘gut dysbiosis’ characterized by reduced beneficial gut bacteria and metabolites and increased pro-inflammatory microbes [112,113]. Moreover, previous studies by our lab demonstrated that gut dysbiosis could pave the way towards gut hyper-permeability (‘leaky gut’), which in turn instigates local and systemic inflammation and impact brain health by inciting neuroinflammation and impaired gut–brain axis [26]. This phenomenon of leaky gut and hyperinflammation are implicated in aging-associated disorders, including type-2 diabetes, obesity, cardiovascular disease, and cognitive impairment [114]. 

**Figure 2:**
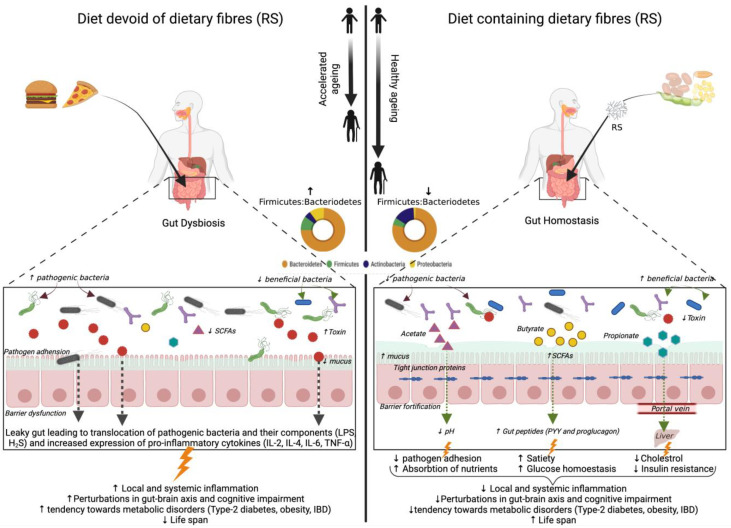
Illustration depicting the reported and purported beneficial effects of resistant starch (RS) on aging-associated gut microbiota and metabolic health. SCFA: short-chain fatty acids; LPS: lipopolysaccharide; IBD: inflammatory bowel disease; IL: interleukin; TNF: tumor necrosis factor; 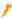: stimulation; ↑: increased; ↓: decreased.

Dietary modulation of gut microbiota composition and metabolites through the supplementation of resistant starch has the potential to extend the health span of the older population by improved gut barrier function and increased expression of gut peptides signaling glucose homeostasis together with lipid metabolism [115]. The beneficial effects of resistant starch (RS) consumption on aging-associated gut microbiota and metabolic health are depicted in Figure 2. However, to date, a limited number of studies related to the effect of resistant starch on aging-associated gut microbiota and health outcomes have been explored and are summarized in Table 4. The effect of RS2 supplementation with a high-fat diet in aged mice models has been studied recently [25]. The study revealed a decreased abundance of *Proteobacteria* and its genus *Desulfovibrio*, the species of which are involved in LPS associated pathogenicity and hydrogen sulfide (H_2_S) production. H_2_S impairs mitochondrial respiration in colonocytes as well as butyrate oxidation that provides energy to cells, thereby promoting inflammation [25]. Other obesity-associated genera viz., *Oscillibacter*, *Lachnoclostridium*, *Tyzzerella, Ruminiclostridum 9,* and *Helicobacteria* are also reduced in this study. *Alistipes*, an aging-associated genus, was decreased in some studies post RS2 supplementation [25,116]. RS incorporation in the diet of aged mice also decreased the abundance of *Parabacteroides* and *Rikenella,* which are usually linked with IBD [25,117]. The depletion of *Bifidobacterium* and *Akkermansia* spp. has been reported as person ages (Collado et al., 2007). RS2 is shown to increase these two taxa in older mice models [116,117]. The fermentation of RS by primary starch degraders is shown to promote the growth of secondary starch degraders like *Allobaculum*, a genus involved in butyrate production [117]. 

Among the SCFAs, butyrate is a key metabolite involved in intestinal homeostasis, enhancement of intestinal barrier functions, and promotion of gut peptides (proglucagon and PYY) involved in satiety [119]. Therefore, its promotion in the distal gut is believed to benefit the elderly, as studied by [25,116]. Peixoto et al. demonstrated increased levels of butyrate, propionate, and total SCFAs in 11.5-year-old dogs after consumption of corn-based RS [120]. It had also been postulated by studies of [116] that mere consumption of RS for a duration of 3 months is not sufficient to significantly relieve aging-associated pro-inflammatory response markers. However, the same can be improved if prebiotics are incorporated in the diets before 70 years of age to prevent the increase of leaky gut-linked inflammatory disorders. The above studies in aged humans and animals cohorts provide a positive correlation of RS consumption in improving gut health and gut microbiota diversity, but the results may also be dependent upon many other physiological factors and may vary between human and animal subjects. 

## 8. Conclusions and Future Prospects

In many regions of the world, dietary pulses fall in the category of neglected staple crops. However, pulses are a good source of high-quality protein and dietary fibers and could offer a cheaper and more sustainable alternative to animal-based protein to address food insecurity concerns. Besides, from a health point of view, several studies discussed herein have highlighted the potential of pulses to positively modulate gut health and to mitigate the risk of various metabolic diseases by beneficially modulating the gut microbiota and strengthening the colonic mucosal environment. The increase or decrease in specific gut microbial signatures after pulse consumption signifies improvement in various diseased states such as obesity, IBD, hypercholesterolemia, and colorectal cancer. 

However, resistant starches derived from dietary pulses remain relatively less explored functional ingredient. Although no clinical studies have been done to date, some of the pre-clinical studies and in-vitro fecal fermentation studies have revealed their potential in modulating the gut microbiome-metabolome arrays and ameliorating several non-communicable gut and metabolic diseases. Likewise, there is a paucity of studies examining the effect of resistant starch on aging-associated diseases except for a few existing studies examining the effect of maize, potato, acorn, and sago-derived RS on gut-associated healthy aging and well-being. Nevertheless, there is ample evidence suggesting that the incorporation of these ‘prebiotic’ food components in a healthy dietary pattern fosters the growth of beneficial gut bacteria and significantly enhances the production of SCFAs in the colon. However, the positive impact of RS on gut health from animal and in-vitro studies may not be directly translated or extrapolated to humans, largely due to considerable differences in the human gut microbial composition among individuals, and disparities in study design, habitual intake, dose and type of RS used during different intervention studies. This presents an exciting opportunity for future research involving rational and stringent design of longitudinal multi-omics clinical nutrition studies addressing the above variables for comprehensively deciphering the detailed mechanisms underlying the effects of different types of pulse-derived RS on the gut microbial ecology, metabolome, intestinal function, and host health. 

Studies to date have focused mostly on cereal- and tuber-derived RS2, whereas concomitant exploration of the effect of different pulses-derived RS types on human microbiome, which remains relatively limited, will certainly advance the knowledge in this field. Manipulation of RS naturally occurring in foods is quite complex owing to numerous intrinsic and external processing factors; therefore, more efforts should be directed to purify the RS from these complex matrices. However, more efforts are also imperative to develop standardized and systemic methods to appropriately characterize chemical composition of purified pulse RS, particular given the substantial structural heterogeneity based on the type of cultivars, method of isolation, interaction with other nutrients, and physical and chemical methods employed for its modifications. Moving forward, the use of purified RS could also further facilitate in drawing more robust conclusions for its use as functional ingredient particularly in the absence of interfering/overlapping effects of other food components such as proteins, polyphenols, other fibers, etc. on the health outcomes. Such studies will also be helpful in formulation and standardization of recommended daily intake dose of RS for various food and nutraceutical applications. Finally, further comprehensive understanding of the detailed structure of different RS types and their selective influence on the diverse gut microbiome structure and functions could further facilitate in the development of microbiome-specific or microbiome-targeted functional foods containing RS within the milieu of healthy dietary patterns.

## Figures and Tables

**Table 1:** Effect of dietary pulses on gut microbiota-related changes in rodents and humans.

Pulse-Type	Cohort	State of Cohort	Age	Dose	Duration of Study	Key Shifts in Gut Microbiota	Outcome	References
Cooked chickpeas	Human	Healthy	18–65 years	200 g/d	3 weeks	Phylum ↑ Bacteroidetes Genus ↑ *Megasphaera* ↓ *Clostridium* I, II, IV, XI clusters Species ↑ *Faecalibacterium prausnitzii* ↓ *Subdoligranulum* ↓ *Clostridium histolyticum, Clostridium lituseburense* groups	Reduction in pathogenic and putrefactive gut bacteria species in cohorts Less intestinal colonization by ammonia-producing bacterial species	[72]
Cooked pinto beans	Human	Healthy; Pre-metabolic syndrome	18–51 years	130 g/d	12 weeks + 4 weeks run-in	Species ↑ *Peptostreptococcus productus* ↓ *Eubacterium limosum*	High propionate production Lower serum total cholesterol, LDL, and HDL	[73]
Cooked navy bean powder	Human	Colorectal cancer survivors (overweight and obese)	47–81 years	35 g/d	28 days	Species ↑ *Clostridium* sp., ↑ *Lachnospira* sp., ↑ *Coprococcus* sp. ↓ *Bacteroides fragilis* ↓ *Anaerostipe* sp.	Boost in microbial richness compared baseline for colorectal cancer survivors but had no effect on their diversity	[74]
Cooked navy beans (incorporated in meals and snacks)	Human	Colorectal cancer survivors (overweight and obese)	NB: 60.9 ± 11.0 years Control: 65.50 ± 3.07 years	35 g/d	4 weeks		Thirty and twenty-six significant metabolite differences in stool samples from baseline and control, respectively Navy bean-derived metabolites (247/560) including N-methylpipecolate, 2-aminoadipate, piperidine, and vanillate Abundance of ophthalmate increased by 5.25 fold	[75]
Beans, chickpeas, peas, or lentils-based foods	Human	Healthy	57 ± 6.3	150 g/d	4 months		Reduction in total cholesterol and LDC by 8.3% and 7.9%	[76]
*Dolichos lablab* L. (standardized extract)	Mice (C57BL/6 male)	IBS model	7 weeks	100–400 mg/kg	15 days		Minimized weight loss with no effect on food intake Attenuated zymosan-induced colonic macroscopic scores Reduced mast cell count, TNF-α in the colon Reduced visceral pain-related behaviors Dose-dependent reduction of c-Fos expression in the brain	[77]
Chickpea supplemented diet	Mice (C57BL/6 male)	Healthy	5 weeks	200 g/kg diet	3 weeks	Family ↓ *Clostridiaceae* (feces only) ↓ *Peptococcaceae* Genus ↑ *Prevotella* ↑ *Dorea* Species ↑ *Ruminococcus flavefaciens* ↓ *Bifidobacterium pseudolongum* ↓ *Parabacteroides distansonis* *↓* Undefined sp. in the *Ruminococcus* genus (cecal only) ↓ *Lactococcus* ↓ *Turicibacter*	Enhanced colon crypt mucus content and mucin mRNA expression Improved expression of epithelial tight junction proteins Enhanced metagenomic functions (e.g., ↑ butanoate metabolism; ↑ flavonoid biosynthesis) Increased SCFAs production Enhanced taxa richness in the cecum	[65]
Cooked white and dark red kidney beans	Mice (C57BL/6 male)	DSS induced colitis	5 weeks	BD + 20% beans	3 weeks		Enhanced acetate, butyrate, and propionate production Increased colon crypt height, and MUC1 and Relmβ mRNA expression Reduced serum levels of IL-17A, TNF-α, IFN-γ, IL-1β, and IL-6	[66]
Cooked Navy bean or black bean	Mice (C57Bl/6 male)	Healthy	4 weeks	Supplementation @20% to the basal diet	3 weeks	Genus ↑ *Prevotella* ↑ *S24-7* ↑ undefined genera within the *Clostridiales* order and *Coriobacteriaceae* family (BB only) ↓ *Oscillospira*, ↓ *Ruminococcus* ↓ *Coprococcus* ↓ *Lactococcus*, ↓ *Streptococcus* ↓ *rc4-4* ↓ *Coprobacillus* ↓ *Parabacteroides* ↓ *Aldercreutzia* *↓* unassigned members o *Peptococcaceae*, *Erysipelotrichaceae*, *Clostridiaceae*, *Mogibacteriaeae*, *Peptostreptococcaceae*, *Christensenellaceae*, *and Rikenellaceae* families Species ↑ *Ruminococcus flavefaciens* ↓ *Ruminococcus gnavus* ↓ *Clostridium perfringens* (NB only) *↓* undefined species in the *Lachnospiraceae* family (BB only)	Enhanced SCFAs production and expression of receptors GPR-41, 43, 109 Increased crypt length, epithelial cell proliferation, goblet cell number, crypt mucus level, and mucin mRNA expression Reduced serum endotoxin concentration Enhanced apical junctional complex components (occludin, JAM-A, ZO-1, and E-cadherin)	[29]
Cranberry beans	Mice (C57BL/6 male)	Healthy and DSS induced colitis	5 weeks	BD + 20% beans	3 weeks	Family ↑ *Prevotellaceae* ↓ *Lactobacillaceae* ↓ *Clostridiaceae* ↓ *Peptococcaceae* ↓ *Peptostreptococcaceae* ↓ *Rikenellaceae* ↓ *Pophyromonadacea* Genus ↑ S24-7 Species ↓ *Ruminococcus gnavus* ↓ *Clostridium perfringens*	*In healthy cohorts:* Increased cecal SCFAs, colon crypt height, crypt goblet cell number, and mucus content Enhanced expression of Muc1, Klf4, Relmβ, and Reg3γ *In diseased cohorts:* Reduced disease severity and colonic histological damage Increased gene expression of barrier function genes (Relmβ, Muc1-3, and Reg3γ) Diminishing of colonic and circulating inflammatory cytokines (IL-1β, IFNγ, IL-6, and TNF-α)	[78]
Lentil, chickpea, bean, and dry pea	Mice (C57BL/6NCrl mice)	Healthy	3–4 weeks	40 g/100 g obesogenic diet (by replacing 35% protein)	17 weeks	Phylum ↑ Bacteroidetes (highest in lentil) ↑ Verrucomicrobia (in bean and lentil) *↓* Firmicutes *↓* Proteobacteria Family ↑ *Muribaculaceae* ↑ *Rikenellaceae* ↑ *Mogibacteriaceae* ↓ *Peptococcaceae* ↓ *Christensenellaceae* Genus ↑ *Allobaculum* ↑ *Sutterella* (II) ↑ *rc4 4* (of Peptococcaceae), ↑ RF32 (of Alphaproteobacteria) ↓ *Oscillospira* ↓ *Dorea* ↓ *Lactococcus* ↓ *Streptococcus* Species ↑ *B. acidifaciens* ↑ *B. pullicaecorum* ↓ *R. gnavus* ↓ *M. schaedleri* ↓ *C. methylpentosum*	High a-diversity, especially for chickpea and dry pea High b-diversity Altered gut microbiota suggestive of anti-obesogenic physiologic outcomes	[18]
Cooked red lentils	Mice (C57Bl/6 male)	Healthy	5 weeks	20% *w/w* basal diet	3 weeks	Phylum ↑ Firmicutes *↓* Bacteroidetes Family ↓ *Parabacteroides* Genus ↑ *Coprococcus* ↑ *Dorea* ↑ *Roseburia* ↑ *Turicibacter* ↑ *Prevotella* ↑ Unknown genus belonging to the *Lachnospiraceae* family	Improved fecal microbiota α-diversity Abundance of SCFA producing bacteria Increased mRNA expression of SCFA receptors (*GPR 41,43),* tight junction proteins (E-cadherin, Zona Occulden-1 Claudin-2)	[64]
Chickpea, lentil, dry peas, and bean	Mice (C57BL/6 male)	Obese	3–4 weeks	40% *w/w* diet	17 weeks	Phylum ↑ Bacteroidetes ↓ Firmicutes (statistically significant in bean and lentil diet) Species ↑ *Akkermansia muciniphila* (bean and lentil fed diet only)	Three fold elevation of bacterial count in the cecum 2.2–5 fold increase in Bacteroidetes to Firmicutes ratio Reduced lipid accumulation in adipose tissue Decreased subcutaneous and visceral fat mass compared to high-fat control but greater compared to a low-fat control 108 differential metabolites identified related to pulse types	[8]
Whole mung bean	Mice (C57BL/6 male)	Diet-induced obesity (1 w HFD feeding)	4 weeks	HFD + 30% bean	12 weeks	Phylum ↑ Bacteroidetes *↓* Firmicutes Family ↑ *Lachnospiraceae* ↑ *Ruminococcaceae* *↓* unassigned member of *Lachnospiraceae* Genus ↑ *Blautia* ↑ unassigned member of *Muribaculaceae* ↑ *Turicibacter* ↑ *Akkermansia* ↑ *Bacteroides* ↑ *Bifidobacterium* ↓ *Ruminiclostridium* ↓ *Mucispirillum* ↓ *Ruminiclostridium* *↓* unassigned member of *Ruminococcaceae* ↓ *Oscillibacter*	Reduction in hepatic steatosis Reduction in body weight gain, fat accumulation, and adipocyte size Significant a- and b- diversity Ameliorated insulin resistance and glucose tolerance Normalization of HFD-induced gut microbiota dysbiosis	[68]
Lentil (*Lens culinaris* Medikus)	Rats (Sprague−Dawley)	Healthy	8 weeks	70.8% red lentil diet	6 weeks	Phylum ↑ Actinobacteria ↑ Bacteroidetes *↓* Firmicutes Family ↓ *Lachnospiraceae* ↓ *Streptococcaceae* Species ↑ *Shutterworthia satelle*	Reduced mean body weight Reduction in body fat and blood plasma triglycerol levels	[79]
Yellow pea flour	Rats	Diet-induced obesity (5 w HFD feeding)	5 weeks	30% *w/w* diet	42 days	Phylum ↓ Firmicutes Species ↓ *C. leptum* (cluster IV)	Attenuated weight gain Low body fat	[70]
Whole yellow pea flour	Hamster (Golden Syrian)	Hypercholesterolemic diet (28 days)	2 weeks	10% replacement of corn starch with pea flour in the diet	28 days	Order ↑ Lactobacillales Genus ↑ Unclassified clostridia ↑ Bacilli	Reduced insulin levels High energy expenditure	[80]

NB: navy bean; BB: black bean; DSS: dextran sodium sulphate; IBS: irritable bowel syndrome; BD: basal diet; ↑: increased; ↓: decreased.

**Table 2:** Effect of dietary pulses-derived resistant starches in modulating gut microbiota and related health outcomes.

RS Type	RS Source	Cohort	State of Cohort/ Sample Type	Age	Dose (g/d)	Duration of Study	Key Shifts in Gut Microbiota	Outcome	References
RS2	Yellow pea	Rats	Diet-induced obesity (5 weeks HFD feeding)	5 weeks	30% *w/w* of AIN-93 M diet	42 days	Genus ↑ *Clostridium* cluster I ↑ *Methanobrevibacter* ↓ *C. leptum* (cluster IV)	Less weight gain than control	[70]
RS2 and RS3	Native and autoclaved-retrograded lentil starch	Mice (BALB/c male)	HFD-induced obesity	Not given; weight 31.86 ± 1.95 g	Intragastric administration @ (400 mg/kg)	6 weeks	Phylum ↑ Bacteroidetes ↓ Firmicutes Genus ↑ Proteobacteria ↑ *Anaerotruncus* ↑ *Bacteroides* ↑ *Bacilli* ↓ *Clostridium* ↓ *Enterococcus* ↓ *Streptococcus* ↓ *Leuconostoc*	Suppression of body and liver weight gain Improvement in serum glucose and lipid profile Enhanced antioxidant status and gut microbiota structure	[37]
RS1	Intact cotyledon cells from pinto bean seeds	Human (N = 3)	Feces	Not given; BMI (18.5–25 kg/m^2^	In vitro fecal fermentation study. 50 mg of intact, weakly damaged, and highly damaged cells added to feces: phosphate buffer solution (1:3 *w/v*); incubated for 24 h		Genus ↑ *Blautia* ↑ *Roseburia* ↓ *Fusobacterium*	Butyrate production increased as cell wall integrity weakens Injection of intact cells has microbiota composition more closely related with the purified cell wall polysaccharides	[19]
RS2 and RS3	Native pea starch and retrograded autoclaved starch	Human (N = 4)	Feces	20–26 years	In vitro fecal fermentation study. 3% resistant starch residues post 8 h simulated gastrointestinal digestion added to basal nutrient medium containing fecal slurry in ratio 1:9; incubated for 24 h		Phylum ↑ Firmicutes ↑ Bacteroidetes ↓ Proteobacteria ↓ Actinobacteria ↓ Verrucomicrobia. Genus ↑ *Bacteroides* ↑ *Megamonas* ↑ *Bifidobacterium* ↓ *Clostridia* ↓ *Fusobacterium*, ↓ *Faecalibacterium* ↓ *Lachnoclostridium*	Significantly higher acetate, propionate, and total SCFAs than control Decreased α- diversity levels of intestinal flora	[86]
RS2 and RS3	Native and pullulanase-debranched and acid-hydrolyzed pea starches	Human (N = 5)	Feces	20–25 years	In vitro fecal fermentation study. 3% resistant starch added to basal nutrient medium containing fecal slurry in ratio 1:9; incubated for 24 h		Phylum ↑ Bacteroidetes ↑ Firmicutes (in debranched and acid hydrolyzed samples) ↑ Actinobacteria ↓ Proteobacteria Genus ↑ *Bacteroides* ↑ *Blautia* ↑ *Collinsella* ↑ *Eubacterium* ↑ *Bifidobacterium* ↑ *Ruminococcus* ↓ *Fusobacteria* ↓ *Escherichia* ↓ *Prevotella*	High propionate concentration followed by acetate and butyrate Reduced diversity index [89] and the richness estimator (Chao index)	[87]
RS1 and RS3	Intact cotyledon cells of pinto beans and heated to different temperatures (60, 80, and 100 C for 1 h)	Human (N = 4)	Feces	20–30 years	In vitro fecal fermentation study. 50 mg of intact, weakly damaged, and highly damaged cells added to feces: phosphate buffer solution (1:3 *w/v*); incubated for 24 h		Genus ↑ *Roseburia,* ↑ *Coprococcus* ↑ *Bifidobacterium* ↑ *Faecalibacterium* ↑ *Blautia* ↑ *Bacteroides* ↑ unclassified *Enterobacteriaceae* ↓ unidentified members of *Lachnospiraceae* Species ↓ *Roseburia faecis*	High acetate followed by butyrate and propionate High a-diversity	[88]

HFD: high-fat diet; ↑: increased; ↓: decreased.

**Table 3:** Effect of dietary cereals- and tubers-derived resistant starches on human gut microbiota.

Sources	RS Type	Dose	Duration	Bacteria ↑ (Genus)	Intervention	References
Beans, wheat, maize, and barley	RS2	22 g + 25 g fiber	4 weeks	*Ruminococcus*	-	[90]
High amylose starch (unspecified)	RS2	40 g/d	4 weeks	*Ruminococcus*	-	[91]
High amylose starch (hi-maize 260)	RS2	45 g/d	12 weeks	-	Prediabetes	[92]
Hylon VII (70%RS)	RS2	30 g + 150 mL milk	6 weeks	*Bacteroides*	Cervical cancer (acute radiation proctitis)	[93]
Raw potato, high amylose starch (hi-maize 260), and Arabinoxylan used in bread rolls and pancakes	RS2	24 g/d, bread rolls (7.0 g/d) and pancakes (8.4 g/d), bread rolls (6.0 g/d), and pancakes (8.4 g/d)	4 weeks (X2)	*Bifidobacterium*	Metabolic syndrome	[94]
High amylose starch (hi-maize 260)	RS2	Diet A: (66 g/d and 4 g/d) Diet B: (48 g/d and 3 g/d)	2 weeks	*Ruminococcus*	Cardiovascular disease (plasma levels)	[95,96]
Novelose 240 and 330	RS2	30 g/d	3 years	-	Hereditary colorectal cancer	[97]
High amylose starch (hi-maize 260) and RDS (unspecified)	RS2	50 g/d (30 g Rs + 20 g RDS)	4 weeks	-	Skeletal muscle and adipose tissue metabolism	[98]
Biscuit (high amylose starch)	RS2/RS3	20 g/d (4 weeks) + 25 g/d (4 weeks)	8 weeks	*Faecalibacterium*	Chronic kidney disease	[99]
Uncooked high amylose corn starch (63.3%RS) and extruded high amylose corn starch (29.9%RS) (Hylon VII)	RS2/RS3	32 g/d + Lithium	4 weeks	-	Colon cancer	[100]
Crackers (RS2: hi-maize 260 (60%RS); RS4: MGP Fiberysn^®^ RW (85%RS))	RS2/RS4	33 g	17 weeks	Bifidobacteria and *Parabacteroides* (RS4), *Ruminococcus* and Eubacterium (RS2)	-	[52]
Bread (tapioca)	RS3	6 g/d	12 weeks	-	Overweight and obesity (post-prandial blood glucose level)	[101]
Unknown source	RS3	50–60 g/d	10 weeks	*Ruminococcus*	-	[102]
Scone (high amylose corn starch (Verafibe^TM^ 2470)	RS4	Unknown	1 weeks	-	Postprandial glycemic response	[103]
Hi-maize 260, Ingredion, USA	RS2	16 g/d	4 weeks	*Roseburia* and *Ruminococcus*	Chronic kidney disease	[104]
High-amylose maize starch acetylated and butylated	RS2	40 g/d	6 weeks	*Bifidobacterium*	Type 1 diabetes	[89]
Crystalline maize, cross-linked tapioca, and cross-linked potato	RS4	35 g/d	-	Crystalline maize (Eubacterium), cross-linked tapioca (*Parabacteroides*), and cross-linked potato (ND)	-	[105]
High amylose wheat	RS2	160 g bread and 75 g biscuits each day	4 weeks	*Roseburia inulinivoran* *Barnesiella intestinihominis* *Alistipes putredinis*	2-wk low dietary fiber run-in period before feeding with RS diet	[106]

Modified from [107]. “-”: unreported; ↑: increased.

**Table 4:** Effect of dietary resistant starches on aging-associated gut microbiota and health outcomes.

RS Type	RS Source	Cohort	State of Cohort	Age	Dose (g/d)	Duration of Study	Key Shifts in Gut Microbiota	Outcome	References
RS2	*MSPrebiotic* from potato	Human	Healthy	Elderly (>70 years) Mid-age (30–50 years)	30 g/d	12 weeks	Phylum ↓ Proteobacteria Genus ↑ *Bifidobacteria* *↑* Prevotella (only in elderly) *↑* Alistipes (only in elderly) Species ↑ *Ruminococcus bromii* (only in mid-age)	Marginal increase in butyrate level in elderly	[116]
RS2	*MSPrebiotic* from potato	Human	Healthy	Elderly (>70 years) Mid-age (30–50 years)	30 g/d	12 weeks		Reduced blood glucose levels and insulin resistance in elderly	[118]
RS2	High-amylose maize	Mice (C57BL/6J male)	Healthy	18–20 mo	18–36% RS	10 weeks	Phylum *↑* Bacteroidetes ↓ Firmicutes Family ↑ *Lachnospiraceae* ↑ *Ruminococceae* Genus ↑ *Bifidobacterium, ↑ Akkermansia* ↑ *Allobaculum* ↓ *Alistipes* ↓ *Parabacteroides*	Increased proglucagon gene expression No significant effect on PYY expression Increase in cecal (empty and full) and entire gastrointestinal tract weights	[117]
RS2	High amylose maize starch with 56% RS2	Mice (C57BL/6 female)	HFD feeding	18 mo	HFD + 20% RS2	16 weeks	Phylum ↓ Proteobacteria Genus ↑ *Ruminococcaceae ↑ Lachnospiracea* ↓ *Desulfovibrio* ↓ *Ruminiclostridium 9* ↓ *Lachnoclostridium* ↓ *Helicobacter* ↓ *Oscillibacter* ↓ *Alistipes* ↓ *Peptococcus* ↓ *Rikenella* ↓ *Marvinbryantia* ↓ *Parabacteroides*	Decreased abundance of pathogen taxa Reversed weight gain and hepatic steatosis and inflammation Increased intestinal permeability Decreased serum and fecal LPS, hepatic IL-4, and colonic IL-2 expression Increased expression of colonic mucin 2 High butyric acid levels and low isobutyric and isovaleric acid levels	[25]
RS2	Acorn and sago	Mice (C57BL/6J male)	HFD induced obesity	8–10 weeks	HFD + 5% RS	8 weeks	Phylum *↑* Bacteroidetes ↓ Firmicutes Genus *↑* S24_7 ↓ *Oscillospira* ↓ *Desulfovibri* ↓ *Bilophila*	Ameliorate HFD-induced glucose intolerance and insulin resistance Increase in SCFAs levels Decrease in leaky gut and inflammation	[26]
RS2	High-amylose maize	Mice (C57BL/6J male)	Healthy	18–20 mo	18–36% RS	10 weeks	-	No effect on body weight and body composition Increased cecum weights Increased expression of cecal proglucagon and PYY mRNA No significant difference in soluble cytokine receptors (sVEGFR1, sTNF-Rb, sIL-4R, sRAGE and sIL- 2Ra) and TNFa expression (gene and protein) in visceral fat	[119]
RS2 + RS3	Corn (having low starch gelatinzation and high RS)	Dogs (Beagles)	Healthy	11.5 ± 0.38 years	Feed supplemented @ 1.46% RS	61 days	-	↑ proglucagon level ↓ fecal pH ↑ fecal butyrate, propionate, and total SCFA concentrations	[120]

“-”: unreported; HFD: High-fat diet; ↑: increased; ↓: decreased.

## Data Availability

Not applicable.
